# The efficacy of a generic doxycycline tablet in the treatment of canine monocytic ehrlichiosis

**DOI:** 10.4102/jsava.v86i1.1193

**Published:** 2015-03-25

**Authors:** Josephus J. Fourie, Ivan Horak, Dionne Crafford, Heidi L. Erasmus, Ockert J. Botha

**Affiliations:** 1 ClinVet International, Universitas, South Africa; 2 Department of Veterinary Tropical Diseases, University of Pretoria, South Africa; 3 Department of Zoology, University of Johannesburg, South Africa; 4 Vetsbrands, Fairland, South Africa

## Abstract

The objective of the present study was to evaluate the therapeutic efficacy of a generic doxycycline tablet (DoxyVet^®^) against *Ehrlichia canis* infection in dogs. Canine monocytic ehrlichiosis is caused by the bacterium *E. canis* and transmitted by the brown kennel tick (*Rhipicephalus sanguineus*). Six disease-free and tick-free dogs were infested with *E. canis*-infected ticks. Once diagnosed (with polymerase chain reaction [PCR] analysis and platelet counts) as positive for infection, doxycycline tablets were administered orally once a day for 20 consecutive days, at a target dose level of 10 mg/kg. The actual dose administered was calculated as ranging between 10 mg/kg and 11.7 mg/kg. The PCR analysis, 28 days after the first administration of the tablets, failed to detect *E. canis* in any of the dogs. On Day 56 of the study, four of the dogs were diagnosed with *E. canis* for the second time and a fifth dog was diagnosed on Day 70. The platelet counts of the sixth dog remained within normal levels and it was discharged from the study on Day 84. Doxycycline tablets were then administered to the remaining five infected dogs for 28 consecutive days. Four of these dogs had no positive PCR results during the following 3 months. The fifth dog was diagnosed with *E. canis* for the third time 58 days after the last tablets of the second treatment had been administered, after which it was rescue treated (doxycycline for a further 28 days). The results indicate that doxycycline administered in tablet form (DoxyVet^®^) at 10 mg/kg – 11.7 mg/kg body mass once daily for 28 consecutive days clears most dogs of infection. The importance of a concomitant tick-control programme is therefore stressed.

## Introduction

Canine monocytic ehrlichiosis (CME) is caused by the gram-negative, obligate intra-cellular bacterium *Ehrlichia canis* and is transmitted to domestic dogs by infected kennel ticks, *Rhipicephalus sanguineus* (Neer *et al*. 2002; Mylonakis *et al*. 2010).

The incubation period lasts anything from 8 to 20 days. During this time the bacteria invade the cytoplasm of circulating monocytes, in which they multiply by binary fission forming intracellular clusters known as morulae, before spreading throughout the body (Neer & Harrus 2006). *Rhipicephalus sanguineus* is the only effective natural vector of *E. canis* (Groves *et al*. 1975; Neer *et al*. 2002); however, *E. canis* can also be transmitted experimentally by the American dog tick (*Dermacentor variabilis*) (Neer *et al*. 2002). Because *R. sanguineus* is found in most parts of the world (Walker, Keirans & Horak 2000), canine ehrlichiosis also occurs in most of the world.

Canine ehrlichiosis may present in three forms: acute, subclinical/subacute, and chronic. These stages or forms may not be easy to distinguish in naturally infected animals, as the spectrum of prominent clinical manifestations may overlap (Mylonakis *et al*. 2010). Clinical signs may include: lethargy, anorexia, fever, swollen lymph glands, weight loss, thrombocytopenia, anaemia, hypergammaglobulinaemia, pancytopenia, haemorrhage and epistaxis (Immelman & Button 1973; Neer & Harrus 2006).

A great deal of confusion currently exists as to the true identity of the vector tick, *R. sanguineus*. Ticks known by this name on different continents are not necessarily morphologically similar (Guglielmone *et al*. 2014). Nevertheless, the authors have chosen to use the epithet *R. sanguineus* when referring to the ticks involved in the present study. *Rhipicephalus sanguineus* is a three-host tick and its natural, if not only hosts, are domestic dogs on which all parasitic stages of development feed (Horak 1982; Walker *et al*. 2000). It does not infest domestic cats or wild canids or felids (Horak *et al*. 2000; Horak *et al*. 2010).

*Rhipicephalus sanguineus* invariably requires man-made structures to successfully complete its life cycle (Bryson *et al*. 2000; Horak 1982) and, as a consequence, it is associated with domestic environments such as homes, apartments, shacks, kennels, sheds, cages or even where dogs are chained (Bryson *et al*. 2000; Horak 1982).

Infection with *E. canis* is acquired by *R. sanguineus* when its larvae, nymphs or adults feed on an infected dog (Groves *et al*. 1975). Infection is transmitted transstadially but not transovarially (Neer & Harrus 2006) and, as a result, the larval offspring of infected female ticks are free from infection (Groves *et al*. 1975). *Ehrlichia canis* may survive for several months in infected ticks (Neer & Harrus 2006); hence, infection can be transmitted by adult ticks that have over-wintered as infected nymphs before moulting to adults in spring. In a household, kennel or regions with a warm climate, more than one life cycle can be completed annually (Horak 1982). Although small numbers of adult ticks may be encountered in autumn and winter, the largest numbers are present in the warmer months, which in South Africa are from October/November to March/April (Horak 1982; Jacobs *et al*. 2001). This window period corresponds with spring, summer and autumn seasons when *R. sanguineus* often exhibits increased activity compared with winter (Aguirre *et al*. 2004).

Doxycycline, a long-acting tetracycline derived from oxytetracycline, is used to inhibit bacterial protein synthesis (Drugbank n.d.); however, it has also been studied as an inhibitor of matrix metalloproteinases (Kogawa & Salgado 2012). Like minocycline, it is lipophilic and can pass through the lipid bilayer of bacteria. Doxycycline reversibly binds to the 30-S ribosomal subunits and possibly the 50-S ribosomal subunits, blocking the binding of aminoacyl transfer ribonucleic acid (tRNA) to the messenger ribonucleic acid (mRNA) and inhibiting bacterial protein synthesis (Drugbank n.d.). Doxycycline is concentrated by the liver in the bile and excreted in the urine and faeces at high concentrations in a biologically active form. The biological half-life reported varies from 12–24 h (Kogawa & Salgado 2012) to 18–22 h. (Drugbank n.d.).

Doxycycline is the drug of choice for the treatment of *E. canis* (Lanza-Perea *et al*. 2009; McLure *et al*. 2010; Schaefer *et al*. 2008), and the recommended dosage in dogs is 5 mg/kg – 10 mg/kg, *per os* for 28 days; it can be administered either once or twice daily (recommended interval 12–24 h) (Neer & Harrus 2006).

In the present study, the therapeutic effectiveness of a generic slow-release doxycycline hydrochloride tablet (DoxyVet^®^) Reg. No. G4070 (Act 36 of 1947), administered at 10 mg/kg *per os* once a day for periods of either 20 or 28 consecutive days, was evaluated against *E. canis* infections in dogs.

## Materials and methods

### Design of study

The study was a single-group design, non-randomised, non-blinded, efficacy evaluation that was conducted on a single group of dogs, with each dog acting as its own control.

### Experimental dogs

Six nondescript, adult, intact, male dogs, weighing between 15.4 kg and 25.0 kg at the time of inclusion on Day −1, were allocated to the present study. Prior to inclusion, the animals were confirmed to be sero-negative for *E. canis*. They were dewormed and tick free and were acclimatised in the test facility for at least 7 days before the commencement of the study. Refer to Table 1 for a summary of frequency and study day of action(s) performed.

**TABLE 1:** Study schedule or tabulated timeline summary to elucidate actions performed on the days indicated.

Actions performed	Days
Acclimatisation	Dogs were housed in the facility at least 7 days prior to Day 0
Artificial infection	Prior to Day 0
Administration of IVP	Days 0 to +19
	Days +58 to +85 (4 dogs)
	Days +73 to +100 (1 dog)
Platelet counts	Prior to infection and Days −1, +14, +28, +56, +63, +70, +71, +77, +84, +85, +86, +99, +100, 113/+114, +127, +128, +139, +155, +169, +170 and +184
Blood collection for PCR	Prior to infection and Days −1, +14, +28, +56, +70, +71, +84, +85, +86, +99/+100, +127, +139, +143, +155, +169 and +184
Blood collection for IFA	Prior to infection and Days −1, +14, +28, +56, +84, +85 and +100
Body weight	Days −1, +58, +70, +100, +113, +114, +157, +169 and +184
Clinical examinations	Prior to infection, Day −1 and Days +3, +14, +28, +56, +71, +84, +85, +86, +99, +100, +113, +114, +127, +128, +139, +155, +169, +170 and +184
Body temperature monitoring	Days 0 to +19
	Days +58 to +84
	Days +73 to +100
	Days +141 to +161

Note: Day 0 was defined as the day that an infection with *Ehrlichia canis* was confirmed by polymerase chain reaction analysis.

IVP, Investigational veterinary product; PCR, polymerase chain reaction; IFA, immunofluorescence assay.

### Housing and care

During study preparation, acclimatisation and until the completion of the first phase of doxycycline treatment (Day 0 – Day 19), the dogs were individually housed in cages that were part of an environmentally controlled indoor animal unit. During study preparation, animals were challenged in infestation chambers and all ticks not attached were collected after the challenge. After feeding for 5 days, the attached ticks were removed by combing.

Adequate biosecurity measures included grease tick traps at all doors, as well as double-sided tape traps against the walls to preclude vertical migration of the ticks. After completion of the initial phase, the dogs, that were now tick free, were moved to an outdoor facility for the remainder of the study. The animals were fed once per day according to the feed manufacturer's recommendation. Food and water were provided in stainless steel bowls in the indoor unit, and food was provided in stainless steel bowls and potable water in cement troughs with ball valves in the outdoor unit. To relieve boredom, the dogs were given ostrich bones on which to chew. Trained personnel, under the supervision of a veterinarian, were responsible for the healthcare of the dogs, and no substances that could have interfered with the activity of the investigational product were administered. Permissible compounds included vitamin and mineral supplements, and sedatives. All aspects of housing and management were in compliance with the South African National Standard (SANS), according to the South African Bureau of Standards (SABS) (2008).

### Challenge with *Ehrlichia canis*-infected ticks

The *R. sanguineus* strain that was used is a laboratory strain maintained and cycled at ClinVet International (Pty) Ltd, Bloemfontein, South Africa. The immature and adult stages of the ticks in the breeding programme are fed on rabbits not previously treated with any acaricides. Immature ticks (nymphs) were infected with *E. canis* by acquisition feeding on a dog with confirmed acute ehrlichiosis. Nymphs were allowed to moult and the adult ticks were confirmed to be infected with *E. canis* by polymerase chain reaction (PCR) analysis. The dogs were all challenged (prior to Day 0) with ticks infected with *E. canis*. The ticks were allowed to feed for 5 days and were then removed by combing. The incubation period ranged between 14 and 18 days.

### Inclusion of animals, clinical parameters and diagnostic tests

Rectal body temperatures were measured daily. When five of the six dogs had temperatures exceeding 39.4 °C, blood was collected from all six animals by venipuncture from the jugular vein of each dog.

For platelet counts, blood was collected in an Ethylene-diaminetetraacetic acid (EDTA) tube and the specimens were sent to Pathcare Veterinary Laboratory, Bloemfontein, South Africa for analyses. Platelet counts were performed on the Advia 2120i Hematology System (Siemens, Deerfield Illinois, USA) with veterinary settings. Peripheral slides were prepared and stained for all samples. Any large clots were recorded and redraw was requested. All slides were reviewed by a qualified technician and any platelet aggregation was reported according to the following rules:

- Scanty platelet clumping observed: 1+ (very little impact), increase analyser value by 20.
- Copious platelet clumping observed: 2+ (platelet clump-ing will impact actual count), increase analyser value by 100.
- Proliferate platelet clumping is observed: 3+ (platelet count is compromised and actual count should read within normal to high limits), increase analyser value by 250.

These estimates were calculated by performing a manual count on peripheral slides, which showed platelet aggregation. For immunofluorescence assay (IFA), blood was collected in a non-anticoagulant tube (serum separator or suitable alternative). Blood specimens were centrifuged at 3000 rpm for at least 10 min at room temperature. The serum specimen was divided into primary and duplicate aliquots, with at least 0.5 mL per aliquot. Both aliquots of the serum specimens for IFA were labelled with study number, specimen identification and collection date, and either aliquot 1 or aliquot 2. Both aliquots were frozen at < −35 °C until assayed for *E. canis* antibodies, using a commercial indirect IFA (MegaScreen FLUOEHRLICHIA canis^®^) test kit (MegaCor Diagnostik, Höerbranz, Austria) performed by the ClinVet International (Pty) Ltd Molecular Laboratory, Bloemfontein, South Africa.

Aliquot 1 was used for IFA analysis. Aliquot 2 was the back-up aliquot. The MegaScreen FLUOEHRLICHIA canis^®^ commercial test kit (MegaCor Diagnostik, Höerbranz, Austria), with *E. canis* antigen slides, plus positive and negative control sera were brought to room temperature prior to use. Test specimens were diluted (1:80) in phospate-buffered saline (PBS) buffer with a pH of 7.4 in a microtiterplate or test tubes. The positive and negative controls were ready for use and were not diluted. Positive and negative control sera were included with each test batch. Diluted sera were individually transferred to a well on the *E. canis* antigen-coated slides and the location was recorded for later reference. Slides were then incubated in a humid chamber for 30 min. After incubation, the slides were flicked off in a basin to remove excess sera. Slides were then gently washed three times with fresh PBS. Thereafter, the slides were removed and gently shaken or tapped to remove beaded PBS from the slides. Without letting the slides dry, the wells were subsequently covered with anti-Dog-IgG-FITC conjugate, and incubated in a humid chamber for 30 min. After incubation, the slides underwent the same washing procedure as in the first wash. Slides were then removed and gently shaken or tapped to remove beaded PBS from the slides and dried only at the back and edges with tissue paper without letting the slides dry. Slides were covered with FluorSave^®^ mounting fluid (EMD Biosciences Inc, San Diego, California, USA) and a coverslip, to be examined under an epi-fluorescence microscope. A positive reaction was determined by comparing test specimens with the positive and negative controls.

For PCR analyses, approximately 3 mL whole blood was collected in an EDTA tube. Approximately 1 mL of blood was taken from the 3 mL EDTA tube and stored in a cryotube in a −80 °C freezer (< −70 °C), which served as a secondary specimen for PCR analysis. The remaining whole blood specimens were transferred to the ClinVet International (Pty) Ltd Molecular Laboratory for analysis. Total genomic DNA was isolated from whole blood specimens using a commercial genomic DNA isolation kit (GeneJET Genomic DNA Purification Kit, Thermo Scientific, Lituania). Polymerase chain reaction entailed the use of primers specific to a region of the *E. canis* dsb gene. Up to 400 ng isolated DNA served as a template for PCR amplification of the target region. The PCR products were analysed using agarose gel electrophoresis and the results were documented. A PCR product of approximately 500 bp indicated the presence of the *E. canis* dsb target region in the sample. Positive, negative, no template, as well as internal amplification controls were included in each run.

*Ehrlichia canis* PCR analysis, using primer sets specific for *E. canis*, were tested for PCR detection sensitivity using an artificially created plasmid DNA-based template (IAC). This template consisted of plasmid DNA, harbouring approximately 130 bp Lambda DNA flanked by the primer sites for the primer set on opposite sites. The PCR detection sensitivity was determined in the presence of 45 ng/µL DNA (Lambda DNA) by adding five-fold serially diluted *E. canis* IAC plasmid DNA to each PCR vessel. The serially diluted DNA represented approximately 9190 copies to < 1 copy of the target per reaction. Reaction setup and thermal cycling were performed as per protocol for *E. canis* detection PCR. Detection sensitivity for the *E. canis* was < 15 copies per reaction. Increased assay sensitivity is to be expected when working with non-supercoiled genomic templates, since PCR amplification efficiency is significantly reduced when using supercoiled plasmid DNA (as performed in the current evaluation described above) (Hou *et al*. 2010). Detection sensitivity of < 2 copies of a serially diluted *E. canis* PCR product per reaction was obtained. However, IAC was used as the sensitivity standard in all PCR assay sensitivity determinations, since it is used as an internal amplification control in all *E. canis* PCR assays performed at ClinVet International (Pty) Ltd (Bloemfontein, South Africa).

Specificity determination was conducted on DNA that was isolated from confirmed cases of *E. canis, Brucella canis, Brucella vogeli* and *Brucella rossi*. Specificity determination indicated that no non-specific or cross-reaction products could be detected between the *E. canis* primer set and the different templates used during the validation. The PCR product was subjected to sequence determination. Analysis of sequence data confirmed that the correct product was amplified from the *E. canis*-positive template. Randomly selected PCR products obtained from routine and ongoing projects were subjected to DNA sequence analysis for confirmation and ongoing validation of the study (all positive sequenced clones exhibited > 99% identity towards the reference sequence).

All six dogs were diagnosed as positive for *E. canis* and were therefore included into the study on Day −1.

From Day −1 to Day 184, clinical examinations were performed at approximately 14-day intervals (and sometimes fewer) as follows:

- Body temperatures were monitored from Days −1 to 19; 58 to 84; 73 to 100 and 141 to 161. Body weights were measured on nine occasions between Day −1 and 184 (Table 1).
- IFA tests were conducted on Day −1 and for 15 days thereafter, and then at 28-day intervals (and sometimes fewer) until Day 100.

### Treatment of *Ehrlichia canis* infections

Upon *E. canis*-positive diagnosis, doxycycline in tablet form (DoxyVet^®^, VetsBrands, South Africa), containing 300 mg of active ingredient, was administered to each dog at a target dose rate of 10 mg/kg body mass once a day for 20 consecutive days (Table 2a). This 20-day treatment period constituted the first Investigational Veterinary Product (IVP) administration phase (Days 0–19).

**TABLE 2a:** Study day (Day) and dose administered to each respective dog during the 20-day administration period.

ID number	Body weight for 20-day administration period (kg)	Dose for 20-day administration period	Administration period (Days)
B2C D1C	16.2	¾ tablet	0–19
DF4 840	15.4	¾ tablet	0–19
DF5 D03	19.4	¾ tablet	0–19
DF6 35C	25.0	1 tablet	0–19
DF7 7BD	18.2	¾ tablet	0–19
B2C A65	19.6	¾ tablet	0–19

Note: Day 0–19 referred to as ‘first phase’ in some in-text discussions.

Safety data sheets, as well as a certificate of analysis, were supplied to confirm that the assay for the active ingredient (doxycycline) was within acceptable limits. The tablets were stored in the chemical store at room temperature and administered by a suitably qualified member of the ClinVet personnel.

The tablets were administered orally, and swallowing was encouraged by holding the dog's mouth closed and stroking its throat. Each dog was observed for evidence of swallowing and licking of its lips, and this was followed by visual inspection of the inside of the mouth. On Days 0–2, dogs received their daily ration of food 30±2 min after administration of the tablets, but because of emesis observed within 2 h after dosing in some of the dogs, this period was reduced to 5 min on Day 3 and on all subsequent days. No dogs expelled any intact or partially intact tablets on any of the administration days.

During the second 28-day IVP administration phase (Days 58–85, Table 2b), as well as rescue treatment of one animal (Days 73–100), the same procedures were followed.

**TABLE 2b:** Study day (Day) and dose administered to each respective dog during the 28-day administration period.

ID number	Body weight for 28-day administration period (kg)	Dose for 28-day administration period	Administration period (Days)
B2C D1C	14.92	½ tablet	58–85
DF4 840	14.89	½ tablet	58–85
DF5 D03	19.77	¾ tablet	58–85
DF6 35C	25.63	1 tablet	58–85
DF7 7BD	19.16	¾ tablet	73–100
B2C A65	No second administration period required	-	-

## Results

### Clinical parameters and diagnostic tests

Body weights over the study period are presented in Table 3. All animals gained weight during this period.

**TABLE 3:** Body weights, as recorded over the study period.

Animal ID	Body weight (kg)		
	Day −1	Day +58	Day +70
B2C A65	19.6	-	22.42
B2C D1C	16.2	14.92	12.72
DF4 840	15.4	14.89	14.35
DF5 D03	19.4	19.77	19.52

-, No bodyweight measurement on that day.

**FIGURE 1:**
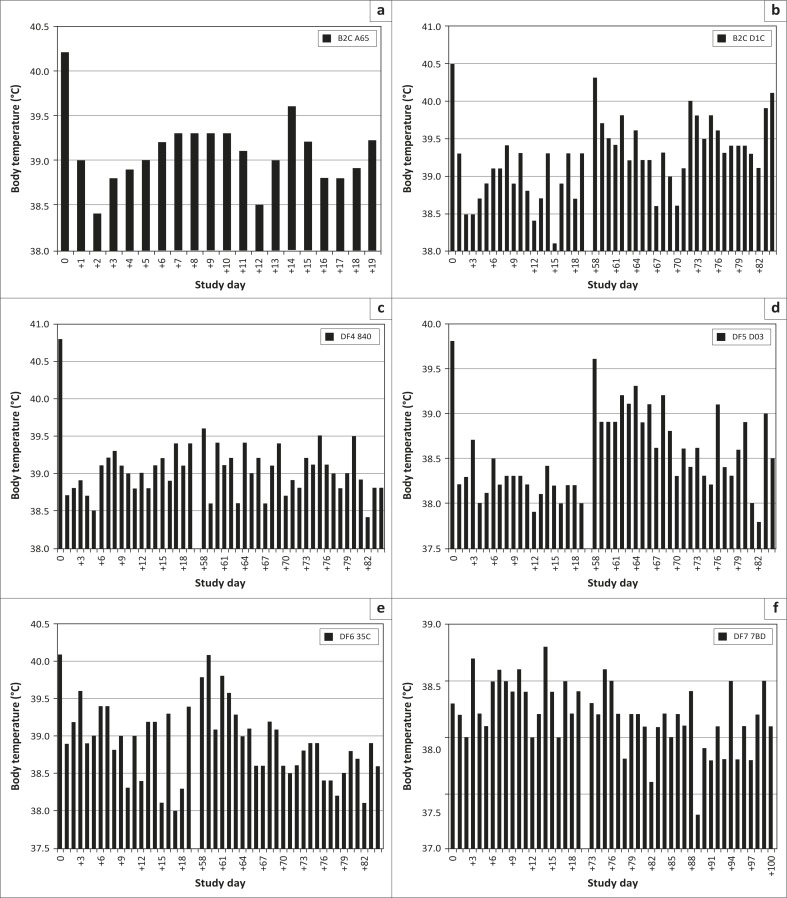
Body temperatures measured for the respective dogs (a–f), on the study days indicated (temperature equal to or exceeding 39.5 °C).

Body temperatures recorded over the study period are presented in Figure 1.

On Day −1, all six dogs for inclusion were diagnosed with *E. canis* infections, as demonstrated by PCR (Table 4) and IFA analysis (Table 5). Elevated body temperatures supported this diagnosis (Figure 1). Treatment commenced on Day 0 and continued to Day 19 (Tables 1 and 2a).

**TABLE 4:** Number of animals for which the polymerase chain reaction results on the respective study days (Days) indicated presence of *Ehrlichia canis* DNA.

PCR results	Positive	Not detected	Number of dogs assessed
Day −1	6	0	6
Day +14	2	4	6
Day +28	0	6	6
Day +56	4	2	6
Day +70	1	0	1
Day +71	0	4	4
Day +84	0	1	1
Day +85	0	4	4
Day +86	0	1	1
Day +99	0	2	2
Day +100	0	1	1
Day +127	0	1	1
Day +139	1	1	2
Day +143	1	0	1
Day +155	1	0	1
Day +169	0	4	4
Day +184	0	1	1

PCR, polymerase chain reaction.

**TABLE 5:** Number of animals for which the indirect immunofluorescence assay results on the respective study days (Days) indicated presence of *Ehrlichia canis* antibodies.

IFA result	Day −1	Day 14	Day 28	Day 56	Day 84	Day 85	Day +100
Positive	6	6	6	6	1	4	1
Negative	0	0	0	0	0	0	0
Number of dogs assessed	6	6	6	6	1	4	1

IFA, indirect immunofluorescence assay.

**FIGURE 2:**
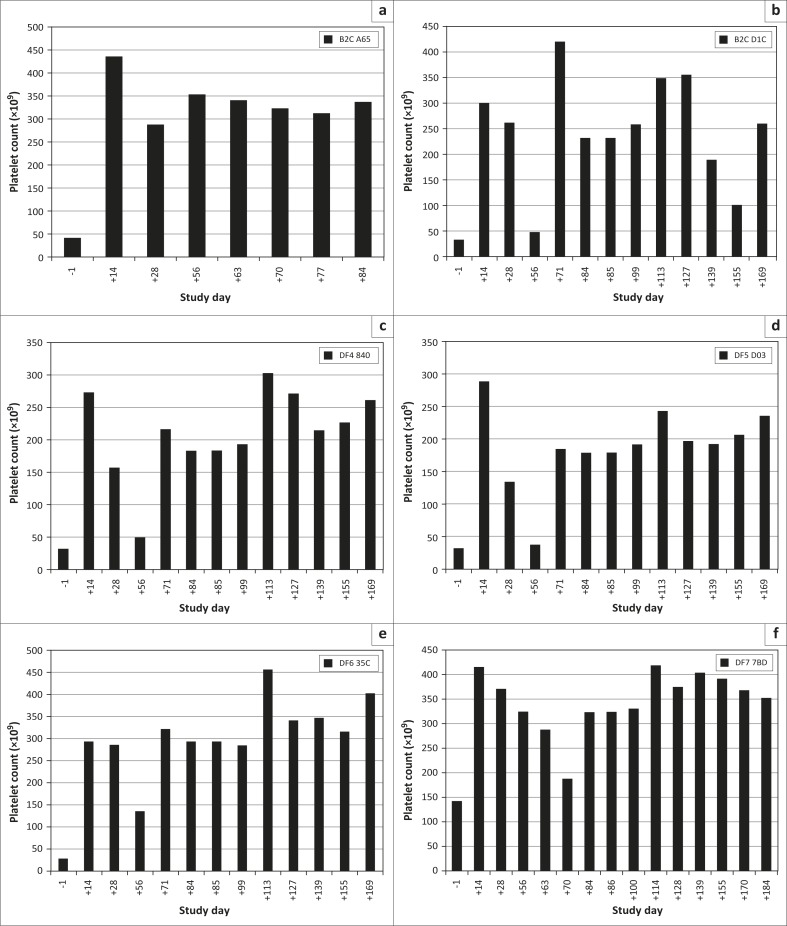
Platelet counts for the respective dogs (a–f), as determined on the study days indicated (a range of 200 × 10^9^ – 500 × 10^9^ is considered to be normal).

On Days 14 and 28, blood collected for PCR analysis (Table 4), as well as for platelet counts (Figure 2), failed to detect *Ehrlichia* in any of the dogs. This was supported by body temperature readings that remained below 39.5 °C (Figure 1) and clinical examinations (Table 6) where no clinical signs consistent with ehrlichiosis were recorded. As could be expected, IFA analyses results (Table 5) did indicate evidence of exposure to *E. canis*.

**TABLE 6:** Abnormal signs observed during clinical examinations and daily observations.

Day	Animal ID	Clinical examination	Daily observations	Comments
−1	B2C A65	Lymph nodes swollen, body temperature 40.5 °C	-	-
14	-	Body temperature 39.6 °C	-	-
−1	B2C D1C	Lymph nodes swollen, body temperature 40.6 °C	-	-
2	-	-	Five times vomitus, yellow mucus	Skin scraping – nothing was found
	-	-	Obvious thinning of hair with crusts at hair base from jawline to jawline (dorsal neck)	-
+3 to +4	-	-	Thinning of hair with crusts – unchanged	-
5	-	-	Thinning of hair unchanged, no crusts	-
+6 to +9	-	-	Thinning of hair unchanged	-
+10 to +15	-	-	Thinning of hair – improving	-
56	-	Lymph nodes enlarged, body temperature 39.9 °C	-	-
+69 to +72	-	-	Dog appears thin	Food increased on Day 72
71	B2C D1C	Thin	-	-
73	-	-	Dog appears very thin	-
+74 to +99	-	-	Thin	-
85	-	Body temp 39.5 °C	-	-
99	-	Thin, body temperature 39.5 °C	-	-
+100 to +107	B2C D1C	-	Very thin	-
+108 to +110	-	-	Thin	-
111	-	-	Thin, improving	-
+112 to +113	-	-	Thin	-
113	-	Body temperature 39.8 °C	-	-
114	-	-	Body condition improving good	-
115	-	-	Body condition unchanged	-
+116 to +124	-	-	Slightly thin	-
−1	DF4 840	Lymph nodes swollen, depressed, body temperature 41.1 °C	-	-
1	-	-	Vomiting once, no tablets, little white powder visible	-
23	-	-	Vomitus, twice, white mucus	-
56	-	Body temperature 39.5 °C	-	-
+69 to +110	-	-	Dog appears thin	-
71	-	Thin	-	-
99	-	Thin	-	-
111	-	-	Body condition improving	-
+112 to +113	-	-	Appears thin	-
114	-	-	Body condition improving good	-
115	-	-	Body condition unchanged	-
+116 to +119	DF4 840	-	Slightly thin	-
+168 to +169	-	-	Slightly thin	-
−1	DF5 D03	Lymph nodes swollen, body temperature 40.3 °C	-	-
2	-	-	Vomitus, once, yellow mucus	-
3	-	-	Small amount of partially digested food vomited, no tablets visible	-
8	-	-	Small amount of partially digested food vomited, no tablets visible	-
69	-	-	Vomitus once, partially digested food	-
70	-	-	Vomitus once, partially digested food and diarrhoea	-
74	DF5 D03	-	Partially digested food vomited, no tablets visible	-
75	-	-	Reddish faeces	-
78	-	-	Vomitus once, mucus	-
−1	DF6 35C	Lymph nodes swollen, body temperature 40.1 °1	-	-
3	-	Salivation	-	-
56	-	Lymph nodes enlarged, body temperature 40.1 °C	-	-
62	-	-	Blood in faeces	-
93	DF6 35C	-	Dark faeces	-
155	-	Body temperature 40.1 °C	-	-
169	-	Body temperature 39.7 °C	-	-
−1	DF7 7BD	Lymph nodes swollen	-	-
6	-	-	Vomitus twice, yellow mucus, no tablets	-
56	-	Popliteal lymph nodes enlarged	-	-
86	DF7 7BD	Popliteal lymph nodes enlarged	-	-
87	-	-	Dog has no appetite	-
100	-	Popliteal lymph nodes enlarged	-	-
114	-	Popliteal lymph nodes enlarged	-	-

Blood sampled on Day 56 for platelet counts (Figure 2) and PCR analysis (Table 4), together with clinical examinations (Table 6), resulted in four of the dogs being diagnosed with ehrlichiosis for a second time. A second administration of doxycycline tablets at 10 mg/kg daily for a period of 28 consecutive days was initiated for the infected dogs from Day 56 onwards (Tables 1 and 2b). These dogs were subjected to platelet counts and PCR analyses on the 14th and 28th day of the 28-day administration period. The PCR analysis did not detect *E. canis* and platelet count results were not indicative of ehrlichiosis.

A fifth dog was diagnosed with *E. canis* on Day 70 (Table 6) and it too was started on the 28-day doxycycline regimen (Tables 1 and 2b). This dog was subjected to platelet counts and PCR analyses on the 14th and 28th day of the 28-day administration period. The PCR analysis did not detect *E. canis* and platelet count results were not indicative of ehrlichiosis.

Platelet counts were performed on the sixth dog at weekly intervals until Day 84, and as these remained within the normal range, this dog was considered cured and was discharged from the study.

Platelet counts and PCR analysis were performed at fortnightly intervals for 3 months after the last treatment on the remaining five dogs. During this time, odd platelet counts below 200 × 10^9^/L were recorded; however, the PCR results of four of these five dogs remained negative, despite intermittent records of platelet counts below 200 × 10^9^/L – 500 × 10^9^/L (Figure 2).

Low platelet counts were also intermittently recorded for the fifth dog as well as a third positive PCR test on Day 139 (Table 4), 54 days after the last day of the second 28-day treatment period. This dog received additional rescue treatment with doxycycline at 10 mg/kg for another 28 consecutive days.

## Ethical considerations

The ClinVet Animal Ethics Committee (CAEC) issued an ‘Ethics approval certificate’, ClinVet International (Pty) Ltd reference CV 13/051, dated 24 July 2013. Dogs were used in this study, since there are no *in vitro* models available that simulate actual *in vivo* use for the evaluation of therapeutic compounds of this type. In addition, study design necessitated use of the target animal.

## Discussion

Doxycycline is the drug of choice for the treatment of *E. canis* (Iowa State University 2013; Lanza-Perea *et al*. 2009; McLure *et al*. 2010; Neer *et al*. 2002; Neer & Harrus 2006; Schaefer *et al*. 2008), primarily because of its superior intra-cellular penetration compared with non-lipid soluble tertracyclines (Neer & Harrus 2006). Treatment is very effective against acute cases, but effectiveness against subacute and chronic cases is considered to be controversial (Mylonakis *et al*. 2010). This statement is exemplified by reports that some chronic cases have been successfully treated after 28 days of doxycycline administration (Eddlestone *et al*. 2007), whilst others demonstrated that not even a 6-week course may be enough to clear infections from sub-clinically infected dogs (Neer & Harrus 2006). However, it must be noted that in the study of Eddlestone *et al*. (2007), the animals were sacrificed at 34–36 days post cessation of treatment. The current experimental work proved conclusively that if a relapse were to occur it would be more than 50 days after the last treatment. The American College of Veterinary Internal Medicine recommends a dosage level of doxycycline of 10 mg/kg for 28 consecutive days as treatment for canine ehrlichiosis (Neer *et al*. 2002). Some sources (Lanza-Perea *et al*. 2009; Mylonakis *et al*. 2010) recommend that the total dosage of 10 mg/kg be divided in two 5 mg/kg doses, administered twice daily.

The initial 20-day period of treatment in the present study, combined with a single daily administration, may possibly account for the fact that five of the six treated dogs again tested positive for *E. canis* some weeks later. The initial treatment period was thus 8 days shorter than the 28 days recommended by Neer *et al*. (2002), whilst the dose was not divided as recommended by Mylonakis *et al*. (2010), amongst others. However, the length of the administration period is probably a more critical variable.

Out of six animals receiving first phase (Day 0–19) treatment, four animals required additional second phase (Day 58–85) treatment before being cleared of infection. A fifth animal also required additional treatment from Day 73 to 100. Harrus *et al*. (1998) applied the same dose rate (10 mg/kg every 24 h) for a period of 42 days and one animal still remained PCR positive, leading to the conclusion that 6 weeks of doxycycline treatment may be insufficient to clear *E. canis* parasites from all sub-clinically infected dogs. The fact that five out of six animals in the present study required treatment periods exceeding 20 days is, thus, not surprising; however, following the second phase (Day 58–85), the four animals in question appeared to be cured.

Harrus *et al*. (2004) treated five experimentally infected dogs with doxycycline hydrochloride (10 mg/kg of body weight every 24 h for 60 consecutive days). They monitored status (blood counts, serology, PCR) up to Day 60 and stated that no ehrlichial DNA could be detected in any of the blood or spleen samples from days 16 post-treatment onwards. Treatment continued up to Day 60, whilst in the present study, first-phase treatment stopped on Day 28. The PCR analyses also indicated no ehrlichial DNA, but recrudescence occurred. It is possible that, should treatment have been discontinued on Day 16 in the study by Harrus *et al*. (2004), recrudescence may have occurred, as was the case in the present study. In addition, they also stated that variable infectious loads may result in longer treatment periods, with reference to naturally infected or chronically infected dogs. Differences in infectious loads may thus explain differences in outcome between these two studies, to some extent. For example, McClure *et al*. (2010) reported that *R. sanguineus* that fed on dogs became PCR positive for *E. canis* after the animals were treated with doxycycline for 28 consecutive days. Furthermore, they stated that this occurred no matter at what stage of the disease treatment was initiated.

Therefore, even when it appears as if treatment has been successful in animals that no longer exhibit any clinical signs of the disease condition, the owner should be warned to observe the dog for several weeks thereafter in case of a recurrence of infection, or fresh infection via bites from infected ticks. The latter is an important consideration, as frequency of tick bite (persistent reinfection) and *E. canis* inoculum size (or infectious load as described by Harrus *et al*. [2004]), may increase the chances for severe/chronic disease (Mylonakis *et al*. 2010).

In southern Africa, co-infection with *Babesia rossi*, the causative organism of canine babesiosis, often confounds a diagnosis of canine ehrlichiosis. Both conditions present with the same abnormal clinical signs, which include an increase in body temperature, lethargy, loss of appetite and anaemia. Furthermore, *Haemaphysalis elliptica* (southern African yellow dog tick, which is the vector of *B. rossi*) and *R. sanguineus* are frequently encountered on dogs examined at the same locality and often on the same dog in such a locality (Jacobs *et al*. 2001; Neves *et al*. 2004). Therefore, particular care must be taken with the diagnoses of tick-borne diseases at these localities; however, it should be borne in mind that *R. sanguineus* also acts as vector for other pathogens apart from *E. canis* that may need to be considered.

Tick control as a prophylactic measure against infection in an *E. canis*-endemic locality is essential. As an example, eight dogs in a laboratory study were fitted with medicated collars with a long-acting acaricidal effect. These animals, together with untreated control dogs, were challenged at 14-day intervals with ticks infected with *E. canis* (Stanneck & Fourie 2013). Any dog that tested positive for *E. canis* on PCR and IFA analyses was removed from the study and replaced with an *E. canis*-negative animal. Not one of the eight collared dogs tested positive for *E. canis* during the 420-day course of the study, whereas 34 of the 35 dogs that were eventually used as controls did (Stanneck & Fourie 2013).

## Conclusion

Generic doxycycline tablets (DoxyVet^®^) at a dose of 10 mg/kg *per os* for 20 consecutive days appears to have successfully cleared six infected dogs of *E. canis*. This was confirmed by the absence of clinical symptoms and by negative PCR, 28 days after treatment had been initiated.

However, after a recurrence of infection in five animals, which was confirmed by PCR, 56 days after treatment was initiated (or 37 days after the end of the first treatment period), the doxycycline tablets successfully reduced parasitaemia beyond the detection limits of the PCR assay after a second administration period of 28 days (48 days in total). Of these five dogs, only one relapsed a month later and was successfully rescue treated with doxycycline at 10 mg/kg *per os* for a further 28 days.

The results indicate that doxycycline, administered in tablet form at 10 mg/kg – 11.7 mg/kg body mass for 28 consecutive days, clears most dogs of infection; however, occurrence of recrudescence necessitates further treatment. It is recommended that the therapeutic protocol of 10 mg/kg doxycycline for 28 days are adhered to, and not shortened to a period of 16 days, as recommended by Harrus *et al*. (2004). Furthermore, the present study demonstrates that this therapeutic protocol may not be 100% effective in treating acute cases and that additional treatment may be required in cases of recrudescence.

Educating dog owners and assisting them with implementing comprehensive acaricidal-prevention strategies, in combi-nation with treatment of *E. canis*, is essential to avoid re-infection, which increases the chances for severe chronic canine monocytic ehrlichiosis.
